# A Novel Innate Response of Human Corneal Epithelium to Heat-killed *Candida albicans* by Producing Peptidoglycan Recognition Proteins

**DOI:** 10.1371/journal.pone.0128039

**Published:** 2015-06-03

**Authors:** Xia Hua, Xiaoyong Yuan, Zhijie Li, Terry G. Coursey, Stephen C. Pflugfelder, De-Quan Li

**Affiliations:** 1 Tianjin Eye Hospital, Tianjin Key Lab of Ophthalmology and Visual Science, Clinical College of Ophthalmology, Tianjin Medical University, Tianjin, China; 2 Ocular Surface Center, Cullen Eye Institute, Department of Ophthalmology, Baylor College of Medicine, Houston, TX, United States of America; 3 Department of Pediatrics, Baylor College of Medicine, Houston, TX, United States of America; Instituto de Salud Carlos III, SPAIN

## Abstract

Fungal infections of the cornea can be sight-threatening and have a worse prognosis than other types of microbial corneal infections. Peptidoglycan recognition proteins (PGLYRP), which are expressed on the ocular surface, play an important role in the immune response against bacterial corneal infections by activating toll-like receptors (TLRs) or increasing phagocytosis. However, the role of PGLYRPs in innate immune response to fungal pathogens has not been investigated. In this study, we observed a significant induction of three PGLYRPs 2–4 in primary human corneal epithelial cells (HCECs) exposed to live or heat-killed *Candida albicans* (HKCA). The C-type lectin receptor dectin-1 plays a critical role in controlling *Candida albicans* infections by promoting phagocytic activity and cytokine production in macrophages and dendritic cells. Here, we demonstrate that dectin-1 is expressed by normal human corneal tissue and primary HCECs. HKCA exposure increased expression of dectin-1 on HCECs at mRNA and protein levels. Interestingly, dectin-1 neutralizing antibody, IκB-α inhibitor BAY11-7082, and NF-κB activation inhibitor quinazoline blocked NF-κB p65 nuclear translocation, as well as the induction of the PGLYRPs by HKCA in HCECs. Furthermore, rhPGLYRP-2 was found to suppress colony-forming units of *Candida albicans* in vitro. In conclusion, these findings demonstrate that dectin-1 is expressed by human corneal epithelial cells, and dectin-1/NF-κB signaling pathway plays an important role in regulating *Candida albicans/*HKCA-induced PGLYRP secretion by HCECs.

## Introduction

Fungal infections of the cornea (fungal keratitis), commonly occurring after corneal trauma, can be sight-threatening without aggressive treatment [1,2]. The innate immune response is a key in the host defense against fungal infections. Corneal epithelial cells, keratocytes, and phagocytes are involved in pathogen-associated molecular pattern recognition leading to a robust innate immune response [3]. In fact, corneal epithelial cells are the first line of host defense and are responsible for immediate recognition and control of microbial invasion [4–7]. Invasive *Candida albicans* (*C*. *albicans*) in the cornea is detected by pathogen-recognition receptors (PRRs) located on the corneal epithelial cells, such as TLRs [8]. PRRs transduce signals that lead to the activation and recruitment of neutrophils and macrophages to sites of fungal infection and enhance the production of antimicrobial factors [9–12].

Peptidoglycan recognition proteins (PGRPs) are a novel family of PRRs that are conserved from insects to mammals [13,14] and function in antibacterial immunity [15]. PGRPs were first discovered in the silkworm Bombyx mori in 1996 as a 19-kDa protein that could recognize peptidoglycans (PGNs) [14]. Drosophila PGRPs are expressed in immune competent cells, such as hemocytes, and are upregulated by PGN [16]. Therefore, it is likely that drosophila PGRPs play a role in insect innate immunity. The Human Genome Organization Gene Nomenclature Committee has renamed PGRPs as peptidoglycan recognition proteins (PGLYRPs) 1–4. Distinct from insects, mammalian PGRPs have direct bactericidal ability since it can recognize bacterial PGN in the innate immune response [17]. All four mammalian PGLYRPs are soluble, intracellular and secreted molecules, which can kill bacteria in the absence of enzymatic activity [18–23]. PGLYRPs have one or two amidase/PGRP domains that bind PGNs, which are major and essential components of the bacterial cell wall [15,24]. In addition to binding PGN, PGLYRPs also bind other microbial cell wall components including lipopolysaccharide, the main component of the outer membrane of Gram-negative bacteria [15,23]. Bactericidal PGLYRP1, PGLYRP3, and PGLYRP4 kill bacteria by a novel mechanism which induces the activation of a stress-response two-component system that leads to lethal membrane depolarization and oxidative stress in bacteria [25]. PGLYRP2 is an N-acetylmuramoyl-L-alanine amidase that hydrolyzes bacterial peptidoglycan resulting in bacterial killing [21,26].

We previously reported that three of the four PGLYRPs can be induced in human corneal epithelial cells (HCECs). This occurs not only in response to PGN extracted from *Staphylococcus aureus*, but also to polyinosine-polycytidylic acid (PolyI:C), a synthetic analog of double-stranded RNA (dsRNA) that is recognized as a molecular pattern associated with viral infection [27]. This finding suggested that PGLYRPs may have a potential anti-viral role in addition to the known anti-bacterial efficacy during corneal infection. Fungal keratitis is often very difficult to treat. The development of new anti-fungal therapies will dramatically improve the clinical outcome for many patients. Therefore, we set out to investigate whether PGLYRPs are induced in HCECs exposed to fungal ligand and could be utilized to improve treatment for fungal keratitis patients. Heat-killed *Candida albicans* (HKCA) contains β-glucan, the major fungal cell wall component, which is recognized by dectin-1 receptor. Here, we present evidence that expression of PGLYRPs increase in HCECs simulated with HKCA. These findings prompted us to explore the potential interaction between dectin-1 and PGLYRPs on HCECs, an important aspect of innate immune response that has not been examined. The purpose of this study was to explore the expression, regulation and signaling pathways utilized by HCECs to produce PGLYRPs in response to *C*. *albicans*/HKCA stimulus.

## Materials and Methods

### Fungus and HKCA

*Candida albicans* strain SC5314, a clinical isolate capable of producing experimental keratomycosis, was cultured on YPD agar (Sigma-Aldrich, St. Louis, MO) for 3 days at 25°C. Colonies were harvested after 3 days of inoculation and diluted in sterile phosphate-buffered saline (PBS) to yield 1×10^6^ colony-forming units (CFU)/μl based on the optical density (OD) at 600 nm, using a predeterminted OD_600_ conversion factor of 1 OD = 3×10^7^ CFU/ml. Dry heat-killed *Candida albicans* (HKCA) was purchased from InvivoGen (San Diego, CA).

### Human Corneal Tissue and Primary HCEC Cultures

Human donors corneoscleral tissues (in 72 hours post-mortem), which did not meet the criteria for clinical use, were obtained from the Lions Eye Bank of Texas (Houston, TX). Human tissues were handled according to the tenets of the Declaration of Helsinki. Donor corneoscleral tissues were cut through the central cornea or peripheral limbus, and frozen sections were prepared as previously described [28,29]. Human limbal epithelial cells were cultured from corneal limbal rim explants as previously described [30,31]. Briefly, each limbal rim was trimmed and dissected into 2 x 2 mm sized explants and cultured in supplemented hormonal epidermal medium (SHEM) containing 5% FBS at 37°C under 5% CO_2_ and 95% humidity. Corneal epithelial cell growth was carefully monitored and culture media was renewed every 2–3 days. Only epithelial cultures without visible fibroblast contamination were used for this study. Upon confluence, corneal epithelial cultures were switched to serum-free SHEM overnight and treated for 2, 4, 8, 16, 24, or 48 hours with a range of concentrations (10^3^–10^6^ cells/ml) of HKCA. Each experiment was repeated at least three times.

### Dectin-1 and NF-κB Signaling Pathway Assay

HCECs were pre-incubated with specific dectin-1 antibodies (10 μg/ml, SC-26094, Santa Cruz Biotechnology, TX), isotype goat IgG (Santa Cruz Biotechnology, TX), Bay11-7082 (10μM) (tlrl-b82, InvivoGen, CA) or NF-κB activation inhibitor (Quinazoline 10μM, Calbiochem, MA, USA) for 1 hour before addition of 10^6^ cells/ml HKCA and incubated for 1, 4, 24, and 48 hours, respectively [32]. HCECs treated with HKCA for either 1 or 4 hours were fixed for immunofluorescent staining to detect NF-κB p65 nuclear translocation. HCECs were treated for 4 hours and subjected to total RNA extraction for measuring PRLYGPs expression by RT and real-time PCR. HCECs were treated for 24–48 hours to be used for immunofluorescent staining.

### Total RNA Extraction, Reverse Transcription (RT) and Quantitative Real-time PCR

Total RNA was extracted from corneal tissues or HCECs using a Qiagen RNeasy Mini kit according to manufacturer’s protocol, quantified by NanoDrop (ND-1000) spectrophotometer, and stored at −80°C. The first strand cDNA was synthesized by RT from 1 μg of total RNA using Ready-To-Go You-Prime First-Strand Beads (GE Healthcare) as previously described [33,34]. The real-time PCR was performed in Mx3005P system (Stratagene) with 20 μl reaction volume containing 5 μl of cDNA that was generated from 50 ng/ml of total RNA, 1 μl of TaqMan Gene Expression Assay primers and probe for human dectin-1 (Hs00224028), PGLYRP-1 (Hs00175475_m1), PGLYRP-2 (Hs00277228_m1), PGLYRP-3 (Hs00364657_m1), PGLYRP-4 (Hs00220648_m1) or GAPDH (Hs99999905_m1), and 10 μl TaqMan Gene Expression Master Mix. The thermocycler parameters were 50°C for 2 min, 95°C for 10 min, followed by 40 cycles of 95°C for 15 s and 60°C for 1 min. A non-template control was included to evaluate DNA contamination. The results were analyzed by the comparative Ct method and normalized by GAPDH, and presented as relative fold change in the expression levels by the treated groups versus untreated controls [31,34].

### Immunofluorescent Staining

The human corneal epithelial cells were fixed with freshly prepared 2% paraformaldehyde at 4°C for 10 minutes. Cell cultures were permeabilized with 0.2% Triton X-100 in PBS at room temperature for 10 min. Indirect immunofluorescent staining was performed as previously described [35,36]. Primary goat polyclonal antibody against human dectin-1 and PGLYRP-2 (SC-50472, Santa Cruz Biotechnology, Dallas, TX), PGLYRP-3 (IMG-391), PGLYRP-4 (MG-414, Imgenex, San Diego, CA), and p65 (622602, BioLegend) were used. Alexa-Fluor 488 conjugated secondary antibodies (R&D Systems) were applied, and propidium iodide (PI) was used for nuclear counterstaining. Secondary antibodies were applied alone were as a negative control and compared to isotype goat IgG. Imaging was completed with a Zeiss laser scanning confocal microscope (LSCM510META, Thornwood, NY).

### Western Blot Analysis

Western blot analysis was performed as previously described [37]. Cytoplasmic and nuclear extracts were prepared using a Nuclear Extract kit (Active Motif) according to manufacturer's instructions. Protein concentration was measured by a BCA protein assay kit. Samples were mixed with 6×SDS reducing sample buffer and boiled for 10 minutes before loading. Equal protein concentrations (20μg/lane) were separated on an SDS polyacrylamide gel and transferred electronically to PVDF membranes. The membranes were blocked with 5% nonfat milk in TTBS (50 mM Tris [pH 7.5], 0.9% NaCl, and 0.1% Tween-20) for 1 hour at room temperature and incubated with primary antibodies to dectin-1 (1:200), PGLYRP-2 (1:200), or β-actin (622102, BioLegend, 1:1000) overnight at 4°C. After three washes with Tris-buffered saline with 0.05% Tween 20 for 10 min each, the membranes were incubated with HRP conjugated rabbit anti-goat IgG (1:1000) or goat anti-rabbit IgG (1:1000, Rockford, IL) for 1h at room temperature. The signals were detected with an ECL Plus chemiluminescence reagent (GE Healthcare), and the images were acquired by a blot imaging station (model 2000R; Eastman Kodak, Rochester, NY).

### *C*. *albicans* Killing Assay by Recombinant Human (rh) PGLYRP-2

In vitro assay of antifungal activity of rhPGLYRP-2 (TP315773, OriGene, Rockville, MD 20850) was performed. *C*. *albicans* at 1×10^4^ CFU were incubated with 400 μl PBS containing different concentrations of rhPGLYRP-2 (0, 1, 10 and 100 μg/ml) at 37°C for different time periods (2, 6 and 24 h). Serial dilution of each reaction mixture were made to inoculate YPD agar plates. Samples (100 μL) were spread evenly over the surface of the plates with sterile glass spreaders. After incubation at 25°C for 72 h, the number of colonies was counted. Experiments were repeated three times.

### Statistical Analysis

Mann-Whitney U test was used to compare the difference between two unpaired groups. One-way ANOVA test was used to make comparisons among three or more groups. Dunnett’s test was further used to compare each treated group with the control group. Statistical significance was determined to be *P* values < 0.05.

## Results

### PGLYRPs 2–4 were induced by *C*. *albicans* in HCECs

**Fig 1:**
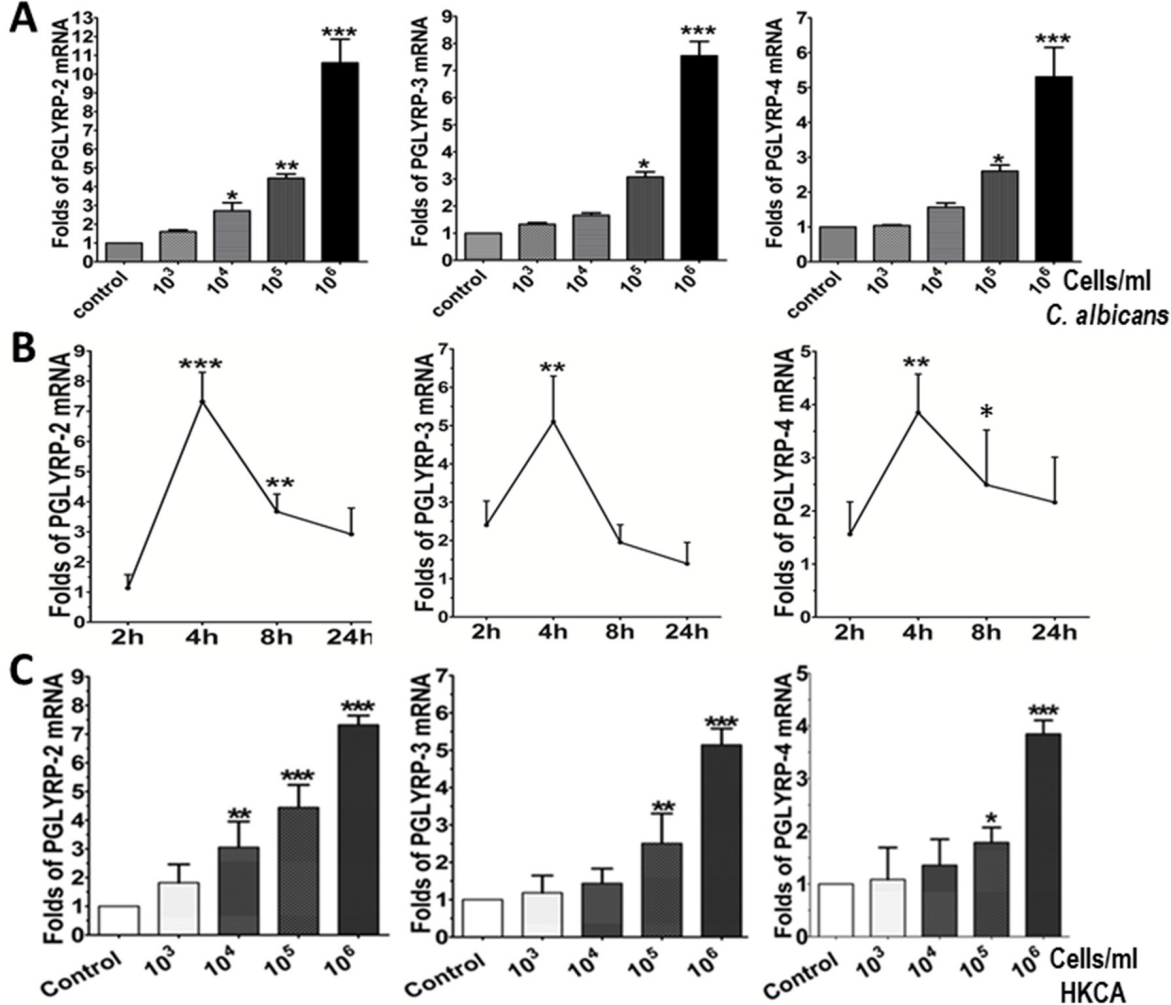
Human corneal epithelial cells (HCECs) produce PGLYRPs in response to live or heat-killed *Candida albicans* (HKCA). Primary HCECs were exposed to live *C*. *albicans* or HKCA with increasing doses (10^3^–10^6^ cells/ml) for 2–24 hours, using untreated cultures as normal controls. A. Dose-dependent stimulation of PGLYRPs 2–4 mRNA in HCECs by live *C*. *albicans* for 4 hours. B. The time course of PGLYRP mRNA induction in HCECs exposed to 10^6^ cells/ml of HKCA, evaluated by RT-qPCR with GAPDH as an internal control; C. Dose-dependent stimulation of PGLYRP mRNA in HCECs by HKCA for 4 hours. Data are presented as mean ± SD, n = 5; * p< 0.05, ** p< 0.01, *** p<0.001, vs. controls.

Primary HCECs were treated with different concentration of live *C*.*albicans* (10^3^–10^6^ cells/ml) for 4 hours to evaluate mRNA expression of PGLYRPs by RT-qPCR. While PGLYRP-1 transcripts were not detected in 45 cycles, PGLYRPs 2–4 were expressed in normal HCECs. The mRNA expression of PGLYRPs 2–4 was significantly increased at dose dependent manner by C. *albicans*. In HCECs treated with 10^6^ cells/ml of live *C*.*albicans*, the mRNA levels of PGLYRP-2, PGLYRP-3 and PGLYRP-4 were induced to 10.60±2.18 (p<0.001), 7.55±0.91 (p<0.001), and 5.31±1.46 (p<0.001) folds, respectively, when compared to the untreated HCECs as controls (Fig 1A).

### PGLYRPs 2–4 were induced in HCECs exposed to HKCA

In order to address the hypothesis that *C*.*albicans* would stimulate the expression of PGLYRPs via dectin-1 mediated pathway, primary HCECs were treated with HKCA, which is known to contains dectin-1 ligand but not TLR2 ligand, at a range of concentrations (10^3^–10^6^ cells/ml) for 4 to 48 hours, and the induction of PGLYRPs was evaluated by RT-qPCR and immunofluorescent staining. Interestingly, the mRNA expression of PGLYRPs 2–4 was significantly induced by HKCA at 10^6^ cells/ml with levels peaking at 4 hours (Fig 1B). PGLYPR-2 expression was significantly lower than PGLYPR-3 and PGLYRP-4 in normal HCECs, but PGLYPR-2 mRNA levels were significantly increased when stimulated by HKCA treatment in a dose dependent manner (7.32±0.97 folds increase in HCECs exposed to HKCA at 10^6^ cells/ml) (Fig 1C). PGLYRP-3 and PGLYRP-4 were constitutively expressed by untreated normal HCECs at higher levels than PGLYRP-2. However, PGLYRP-3 and PGLYRP-4 expression was significantly induced in HCECs exposed to HKCA (10^5^–10^6^ cells/ml) over basal untreated levels. The mRNA expression of PGLYRP-3 and PGLYRP-4 increased to 5.14±1.15 (p<0.001) and 3.85±0.72 (p<0.001) fold, respectively, with HKCA treatment (10^6^ cells/ml). To confirm these results we examined protein expression using immunofluorescent staining (see below).

### Dectin-1 expression increased in human corneal tissue and primary HCECs exposed to HKCA

**Fig 2:**
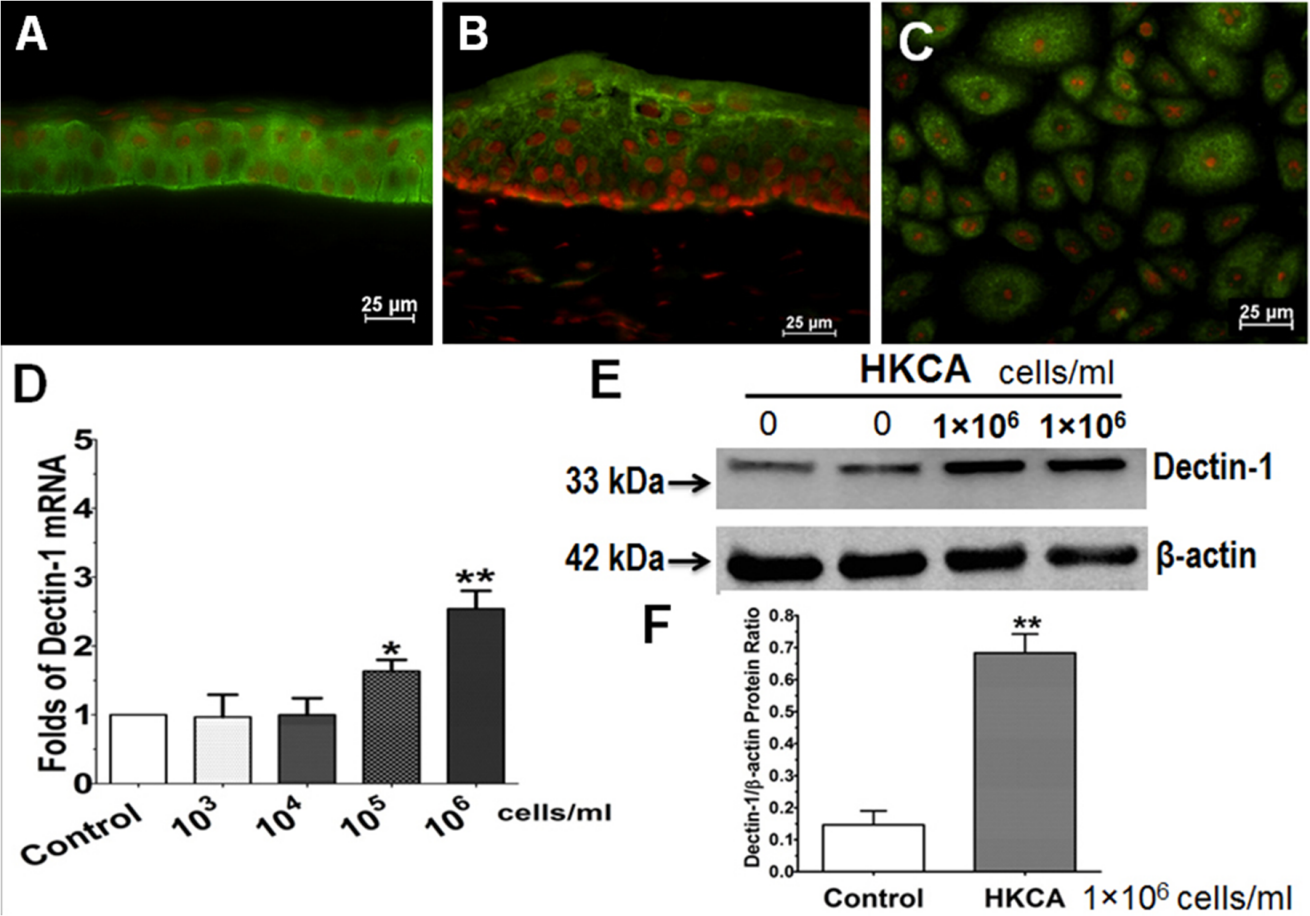
Dectin-1 expression in corneal epithelial tissue and primary cultured HCECs. Representative images showed immunofluorescent staining of dectin-1 on human corneal (A) and limbal tissue (B), as well as in HCECs (C). D. Dose-dependent stimulation of dectin-1 mRNA in HCECs by HKCA for 4 hours. E. Total protein of HCECs treated for 48 hours was extracted with RIPA buffer for western blot with dectin-1 or β-actin antibody. F. Quantitative ratio of the dectin-1/β-actin protein, evaluated by western blotting, in HCECs with or without exposure to 10^6^ cell/ml of HKCA. Propidium iodide (PI) was used as nuclear counterstaining (red color). Magnification: 400Х (bar = 25μm). Data are presented as mean ± SD, n = 5; * p< 0.05, ** p< 0.01, vs. controls.

Dectin-1 is a 28 KDa type II transmembrane protein. It was originally identified as a dendritic cell surface molecule, hence named “Dendritic cell-associated C-type lectin-1” [38]. Recently, studies have shown that dectin-1 is expressed by a variety of myeloid cells, including macrophages, dendritic cells, and neutrophils [39]. There have been few reports showing dectin-1 expression and its response to the fungal ligand by primary human corneal epithelial cells. Immunofluorescent staining indicated that dectin-1 localizes to human corneal tissues and primary HCECs. As shown in Fig 2A & 2B, dectin-1 was localized throughout all corneal epithelium, and extends to superficial layers of limbal region epithelium. In primary HCECs (Fig 2C), dectin-1 was found to be localized to the cell membrane and cytoplasm. Dectin-1 mRNA expression was significantly induced to 2.54±0.78 fold in primary HCECs exposed to HKCA at 10^6^ cells/ml when compared to untreated normal HCECs (Fig 2D, p<0.01). Using a specific antibody to human dectin-1, the protein was detected by western blot in normal HCECs and was significantly increased in HCECs exposed to 10^6^/ml HKCA (Fig 2E). The dectin-1/β-actin protein ratios were significantly increased in HCECs exposed to HKCA compared to normal HCECs (Fig 2F).

### Dectin-1 is required for induction of PGLYRPs by HKCA in HCECs

**Fig 3:**
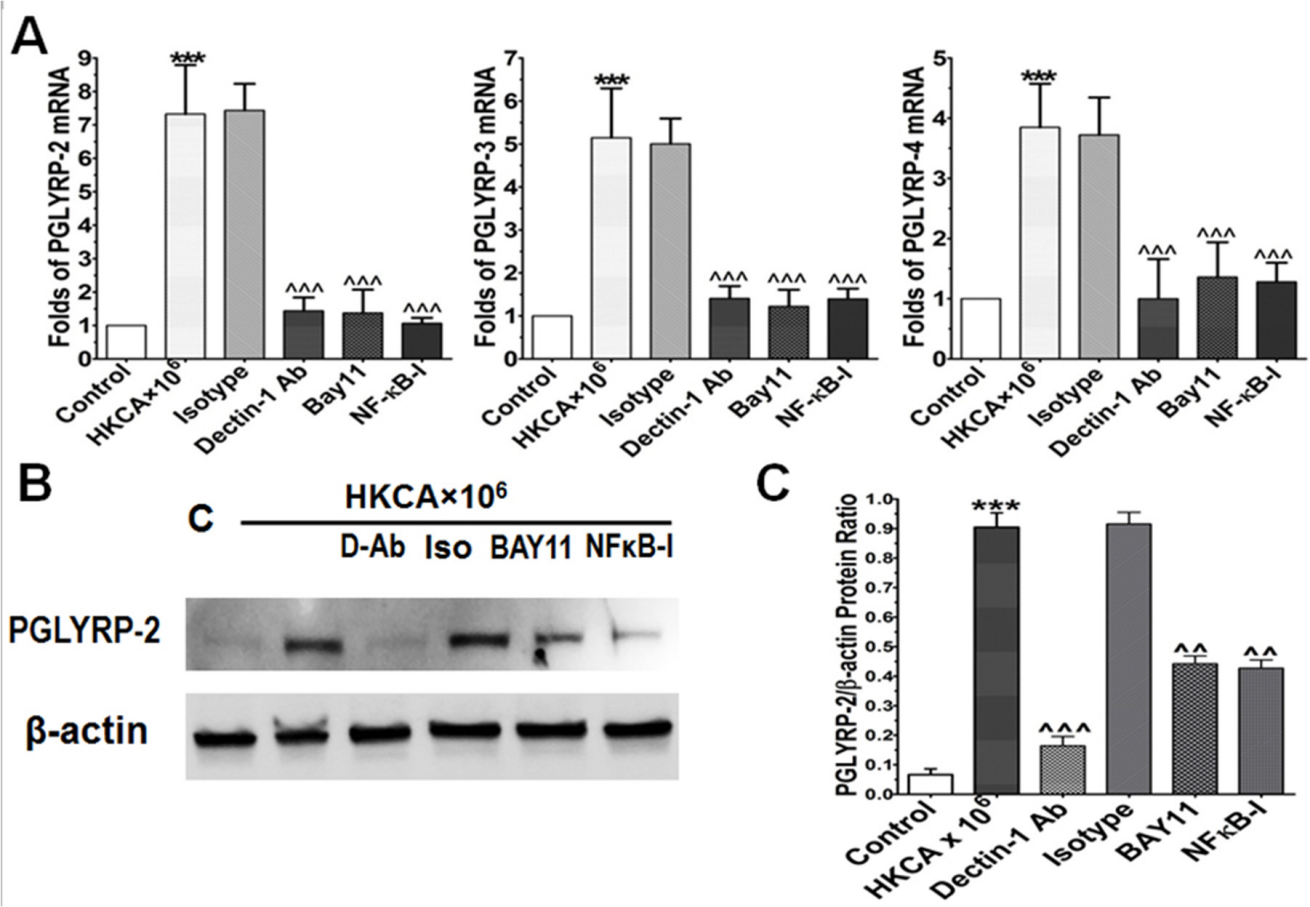
Dectin-1 and NF-κB signaling pathways involve PGLYRPs induction in HCECs exposed to HKCA. A. The HCECs were exposed to 10^6^ cells/ml HKCA with prior incubation in the absence or presence of isotype IgG (10μg/ml), dectin-1 Ab (10μg/ml), BAY11-7082 (10μM) or NF-κB activation inhibitor quinazoline (NF-κB-I, 10μM) for 1 h. Cultures treated by HKCA for 4 h were subjected to RT-qPCR to measure mRNA. B. Total protein of HCECs treated for 48 hours was extracted with RIPA buffer for western blot to examine PGLYRP-2 production. C. Protein levels of PGLYRP-2 were evaluated by western blot using β-actin as control with quantitative ratio of PGLYRP-2/β-actin. Results shown are the mean ± SD of four independent experiments; *** p<0.001, as compared with normal control; ^^ p<0.005, ^^^ p<0.001, as compared with HCECs exposed to HKCA. Magnification: 400Х (bar = 25μm).

**Fig 4:**
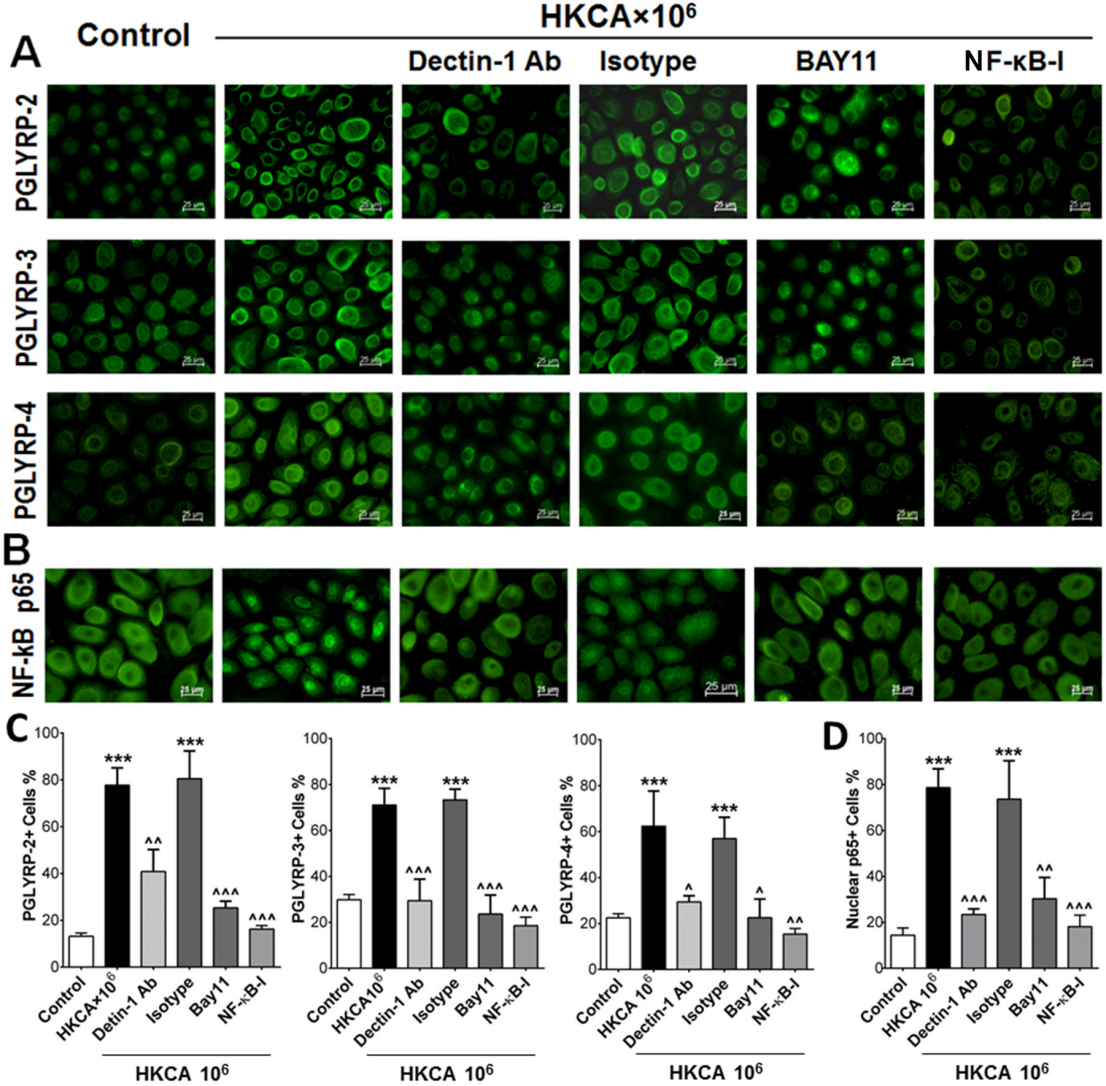
NF-κB p65 activation was induced by HKCA and inhibited by dectin-1 neutralizing antibody and NF-κB activation inhibitor quinazoline (NF-κB-I) in HCECs. A. HCECs were exposed to HKCA (10^6^ cells/ml) with prior incubation in the absence or presence of isotype IgG (10μg/ml), dectin-1 neutralizing Ab (10μg/ml), BAY11-7082 (10μM) or NF-κB activation inhibitor quinazoline (NF-κB-I, 10μM) for 1 h. HCECs were treated with 10^6^ cells/ml HKCA for 48 hours in 8-chamber slides and examined by immunofluorescent staining for PGLYRPs 2–4. B. HCECs were treated for 4 hours in 8-chamber slides and were fixed in acetone for immunofluroscent staining total p65 (nuclear translocation) (green). C. The percentages of positive cells of PGLYRPs 2–4 staining in HCECs in A was quantified. D. The percentages of NF-ĸB p65 nuclear staining positive cells in B was quantified. Images are representatives from three independent experiments. Results shown are the mean ± SD of four independent experiments; *** p<0.001, as compared with normal control; ^^ p<0.005, ^^^ p<0.001, as compared with HCECs exposed to HKCA. Magnification: 400Х (bar = 25μm).

In order to test the hypothesis that dectin-1 is needed for induction of PGLYRPs in HCECs in response to HKCA, we pretreated HCECs with 10 μg/ml of dectin-1 neutralizing antibody or isotype goat IgG for one hour prior to adding 10^6^ cells/ml of HKCA. Dectin-1 neutralization significantly suppressed the mRNA levels of PGLYRP-2, PGLYRP-3, and PGLYRP-4 to 1.44±0.41, 1.41±0.29, and 1.2±0.6 fold, respectively (Fig 3A, p<0.001, n = 4), compared to isotype IgG. Quantitative analysis of western blot for PGLYRP-2 (normalized by β-actin control) confirmed the stimulation effect of PGLYRP-2 by HKCA (p<0.001) and the inhibition of PGLYRP-2 by dectin-1 neutralizing antibody (p<0.001) (Fig 3B & 3C). The inhibitory effect of dectin-1 antibody in HKCA-induced PGLYRP-2 was further confirmed by immunofluorescent staining (Fig 4A).These results suggest that HKCA stimulates the expression and production of PGLYRPs via dectin-1 receptor, which is expressed on corneal epithelial cells.

### NF-kB pathway was involved in dectin-1 mediated induction of PGLYRPs in HCECs exposed to HKCA

The NF-κB transcriptional pathway is an essential component in immune response to infection [40]. Here we further investigated whether the NF-κB signaling pathway is involved in the production of PGLYRPs in HCECs stimulated by HKCA. As shown in Fig 4A, the immunoreactivity of PGLYRP-2 was weaker than PGLYRPs 3–4 in the cytoplasm of untreated primary HCECs. The intensity of all three PGLYRP molecules increased in the cells exposed to HKCA (10^6^ cells/ml) for 48 hours. However, preincubation with the NF-κB activation inhibitor quinazoline (NF-κB-I, 10μM) or IκB-α inhibitor Bay11-7082 (10μM) was found to significantly reduce mRNA expression (Fig 3A) and protein production (Fig 4A) of PGLYRPs -2, -3, and -4 by HKCA (10^6^ cells/ml) in HCECs. These results suggest that PGLYRPs production in HCECs stimulated by HKCA occurs via NF-κB activation. The highly inducible nature of PGLYRP-2 led us to confirm this by western blots. Protein levels of HKCA-stimulated PGLYRP-2 were significantly reduced by treatment with dectin-1 antibody (p<0.001), BAY11 (p<0.005) and NF-κB-I (p<0.005) (Fig 3B & 3C). NF-κB activation was confirmed by immunofluorescent staining, showing the translocation of NF-κB p65 protein from cytoplasm to nucleus in HCECs exposed to HKCA (10^6^ cells/ml) (Fig 4B). Interestingly, the HKCA stimulated p65 nuclear translocation was significantly inhibited by dectin-1 antibody treatment (dectin-1 Ab, 10μg/ml), but not by isotype IgG. Additionally, p65 activation was also inhibited by Bay11 and NF-κB-I (Fig 4B). These results showed that the HKCA stimulated the HCECs and leaded to its NF-κB activation through dectin-1. NF-κB signaling pathway was involved in PGLYRP induction by corneal epithelial cells in response to fungal stimulation.

### *In vitro* anti-fungal activity of rhPGLYRP-2

**Fig 5:**
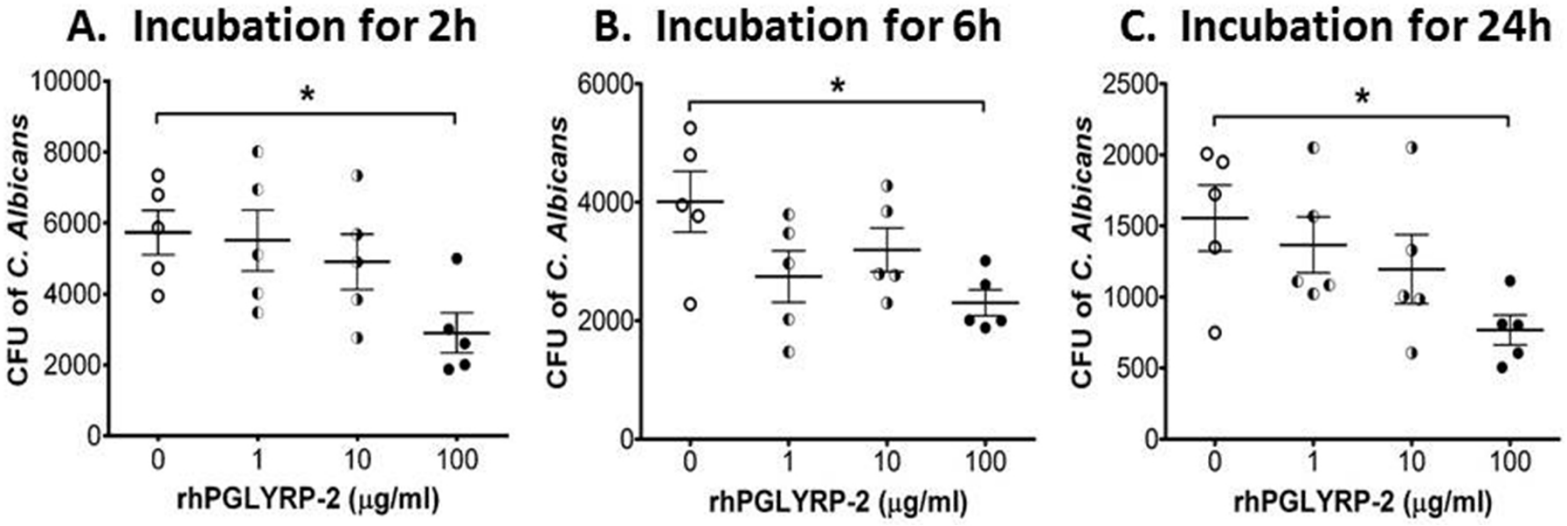
In vitro anti-fungal activity of rhPGLYRP-2. *C*. *albicans* (1×10^4^ CFU) in 400 μL PBS were incubated without or with rhPGLYRP-2 in different concentration (1, 10 or 100 μg/ml) at 37°C for different time periods, 2h (A), 6h (B) and 24h (C). At the end of incubation, the samples were subjected to fungal culture and plate-colony counting. Results were presented as colony-forming units (CFU) of *C*. *albicans* (mean± SD) in cultures with different concentrations of rhPGLYRP-2. Each symbol represents an individual sample, and data are representative of three independent experiments.*p<0.05.

PGLYRP-2 has been reported to have the ability to kill bacteria. To test potential anti-fungal activity of PGLYRP-2, we performed an in vitro *C*. *albicans* killing assay without or with different concentrations of rhPGLYRP-2 (0, 1, 10, 100 μg/ml). As shown in Fig 5A, the colony-forming units (CFU) of *C*. *albicans* at 2 hours were decreased almost 50% to 2901±1261 by rhPGLYRP-2 at 100μg/ml, when compare to 5736±1410 CFU in PBS controls (p<0.05). However, rhPGLYRP-2 at low concentrations (1 and 10 μg/ml) did not inhibit the CFU of *C*. *albicans* growth even with prolonged incubation time for 6 and 24 hours (Fig 5B and 5C). It suggests that PGLYRP-2 may have potential anti-fungal activity to suppress or kill *C*. *albicans*. Further studies are important to investigate whether PGLYRP-2 may increase the resistance of corneal epithelial cells to *C*. *albicans* infection.

## Discussion

Epithelial cells of the ocular surface are the gatekeepers of innate immunity in response to fungal pathogens. Certain TLRs and C-type lectin receptors (CLRs) on epithelial surfaces recognize *C*. *albicans*, and initiate protective responses against pathogens [41]. Cell-surface recognition of fungi produces a cell signaling cascade resulting in a number of growth factors, cytokines/chemokines, antimicrobial peptides, and cell matrix proteins by epithelial cells [9,10,42,43]. Human PGLYRPs are differentially expressed in various organs and tissues. PGLYRP-1 is highly expressed in the bone marrow and granulocyte granules [19,44,45]. PGLYRP-2 is constitutively produced in the liver [19,21] and induced in epithelial cells by exposure to bacteria and cytokines [46]. PGLYRP-3 and -4 are selectively expressed in the skin epidermis, eyes, salivary and sebaceous glands, throat, tongue, esophagus, stomach and intestine. Recent studies have reported that PGLYRP family members are involved in immune modulation in innate immunity [24,47–50]. Several previous reports, including ours, have shown that human corneal epithelium produces PGLYRPs via TLR pathways in response to bacterial or viral components. However, few studies have investigated the PGLYRPs response to fungal infection in corneal epithelial cells. The present study provides evidence that PGLYRP-2, -3, and -4 are induced and differentially regulated in HECEs exposed to *C*. *albicans* and HKCA, indicating that PGLYRPs participate in the corneal epithelial cell mounted defense against fungal invasion.

TLR2 and TLR4 are known to be involved in the host innate immune response to fungal infection [8]. Perhaps the most important PRRs for fungal recognition are CLRs. CLRs comprise a family of six cell-surface proteins including dectin-1. Dectin-1 recognizes β-(1, 3) glucan, which is a major component of the fungal cell wall [51,52]. It is generally accepted that macrophages primarily detect fungi via dectin-1. Binding of dectin-1 induces phagocytosis through a cytoplasmic domain which initiates cell signaling and activation [53,54]. Mice deficient in dectin-1 have impaired inflammation and are more susceptible to fungal infections, demonstrating that dectin-1 is important in the protective immunity in host defense against fungi [55,56].

To date, a majority of studies exploring the role of dectin-1 have focused on expression in macrophages resulting in a classical model of a dectin-1-mediated pathway for detecting fungal infection. Our work here suggests that dectin-1 also functions as a PRR expressed on mucosal surface epithelia and initiates an innate immune response to HKCA. Human Dectin-1 is a small type II transmembrane receptor with a single extracellular carbohydrate recognition (Lectin-like) domain and immunoreceptor tyrosine activation motif in its cytoplasmic tail. We have detected that Dectin-1 immunoreactivity was located at cell membrane and cytoplasm of human corneal epithelium tissue and cultured cells by immunofluorescent staining (Fig 2A–2C) with a goat polyclonal antibody, which was raised against a peptide mapping near the N-teminus located in its cytoplasmic tail. Dectin-1 initiates the innate immune response by inducing the production of IL-1α, IL-8 and defensins in human keratinocytes [57]. Interestingly, we found the production of PGLYRPs was inhibited by dectin-1 neutralization in primary HCECs exposed to HKCA, suggesting another layer of innate immunity in which dectin-1 plays an essential role in inducing PGLYRPs by the corneal epithelium.

Human corneal epithelial cells directly recognize microbial threats and elicit appropriate inflammatory and anti-microbial responses through innate immune recognition receptors. These receptors activate pro-inflammatory transcription factors such as NF-κB, trigger phagocytosis and microbicidal function, and ultimately shape the development of the adaptive immune response. The NF-κB signaling pathway appears to mediate mucosal epithelial inflammation [58,59]. Our data suggests that NF-κB activation is induced by HKCA in HCECs. This is consistent with the other *in vitro* evidence that NF-κB is activated in epithelial cells of *C*. *albicans* infection [60]. *C*. *albicans* epithelial infections suggest that NF-κB could be activated either through triggering of receptors by fungal components or products [61,62]. Dectin-1 has been reported to induce NF-κB activation and cytokine production using particle β-glucan or live fungi in phagocytes [63], but has not been reported to be expressed on corneal epithelial cells. Neutralization of dectin-1inhibited NF-κB activation to the same extent as the IκB-α and NF-κB activation inhibitors did. Overall, this suggests that the innate immune response to fungus in corneal epithelial cells is initiated through dectin-1/ NF-κB signaling. NF-κB activation promotes the expression of relevant inflammatory genes and further mobilizes the immune system to achieve its antifungal effect [12]. Our study indicates that a potential signaling pathway exists that involves the interaction of HKCA and dectin-1 in the induction of PGLYRPs in HCECs. Furthermore, neutralization of dectin-1significantly attenuated the production of PGLYRPs in HCECs. Similarly, the production of PGLYRPs was significantly inhibited by the addition of quinazoline or the IκB-α inhibitor, BAY11. These data demonstrated that HKCA-stimulated PGLYRPs production results from the activation of dectin-1/the NF-κB signaling pathways. Previous studies from our lab found PGLYRPs can be induced by TLRs ligand in HCECs [27], suggesting possible crosstalk within both signaling pathways. Whether dectin-1 collaborates with other receptors to induce PGLYRPs production in response to HKCA in HCECs requires further investigation.

In summary, we have demonstrated that human corneal epithelium expresses three PGLYRPs which can be stimulated by HKCA through the dectin-1/NF-κB signaling pathway. These findings suggest that PGLYRPs participate in antimicrobial defense by contributing to the innate immune response in human corneal epithelium during fungal keratitis.
